# Effects of Different *Daqu* on Microbial Community Domestication and Metabolites in Nongxiang Baijiu Brewing Microecosystem

**DOI:** 10.3389/fmicb.2022.939904

**Published:** 2022-06-30

**Authors:** Fengjiao Mao, Jun Huang, Rongqing Zhou, Hui Qin, Suyi Zhang, Xiaobo Cai, Chuanfeng Qiu

**Affiliations:** ^1^College of Biomass Science and Engineering, Sichuan University, Chengdu, China; ^2^National Engineering Laboratory of Clean Technology for Leather Manufacture, Sichuan University, Chengdu, China; ^3^National Engineering Research Centre of Solid-State Brewing, Luzhou, China; ^4^Lu Zhou Lao Jiao Co., Ltd., Luzhou, China

**Keywords:** Nongxiang Baijiu, simulated fermentation, *Daqu*, pit mud domestication, metabolites

## Abstract

The quality and yield of the fresh Baijiu mainly depend on the activity of pit mud (PM) and the quality of *Daqu*. However, the cultivation of PM is a long-term process, and high-quality *Daqu* can change the community structure of fermented grain (FG) and accelerate the evolution of PM communities. The present research aimed to investigate the four different types of *Daqu* on the FG-fermenting microbial community structure and metabolites and their interphase interactions with PM. These results show that *Kroppenstedtia* in the bacterial community of Taikong *Daqu* (TK) was positively correlated with ethyl caproate, which significantly increased the content of FG volatile metabolites, especially lipid components, and facilitated the accelerated evolution of *Methanobacteriales* and *Methanosarcinales* in PM. *Bacillus* has a high relative abundance in Qianghua *Daqu* (QH), which shows obvious advantages to improving the alcoholic strength of FG and contributing to increasing the abundance of *Methanomicrobiales* in PM. Qianghua and traditional-mixed *Daqu* (HH) have a similar bacterial composition to QH and a similar fungal composition to traditional *Daqu* (DZ), and thus also showed the advantage of increased yield, but the volatile flavor metabolites produced were not as dominant as DZ. β-diversity analysis showed that in TK fermentation systems, FG is more likely to domesticate the structure of PM microorganisms. These results indicated that the interaction between microbial communities in Baijiu fermentation niches was significantly influenced by different *Daqu*. It can not only enhance the key volatiles in FG but also accelerate the evolving direction of the community in PM. *Daqu* fortified by functional genera or microbiota can evolve a community structure more suitable for Baijiu fermentation. The microbiota composition and interaction between the communities in both *Daqu* and PM significantly impacts the yield and quality of the base liquor.

## Introduction

Nongxiang Baijiu (NXBJ) is one of the four famous distilled spirits in China, which enjoys a good reputation because of its unique fermentation process, good taste, and typical flavor characteristics (Zheng and Han, 2016). NXBJ is the top-selling spirit, accounting for approximately 70% of total Baijiu consumption in China (Liu M. K. et al., 2017). For NXBJ production, medium-temperature *Daqu* is an indispensable starter and the quality is closely related to the unique flavor profile and yield of fresh Baijiu. NXBJ is normally fermented using sorghum alone or a mixture of corn, rice, wheat, peas, millet, and sorghum as the raw materials (Zheng and Han, 2016). The streamed mixture-added *Daqu* is loaded into a rectangular cellar (called a mud pit) lined with the PM and fermented anaerobically at ambient temperature for 2∼9 months (Lu et al., 2018). The mixture composed of fermented and fresh grains is steamed under normal pressure conditions by a solid-state pattern, in which the steamed grains, distillation, rectification, and extraction of fresh Baijiu liquor aroma are performed simultaneously. Microorganisms involved in the fermentation process mainly originated from raw materials (Zhang et al., 2019), *Daqu* (Yang et al., 2021), PM, FG, and the environment (Du et al., 2019). The quality and yield of the fresh Baijiu have mainly lied on the activity of PM and the quality of *Daqu* in addition to the fermentation process parameters (Xiao et al., 2017; Wang Z. Y. et al., 2018). Therefore, it has become a research hotspot to produce *Daqu* with better fermentation functions (Fan et al., 2018b; He et al., 2019a; Huang et al., 2021). The main functional microorganisms in *Daqu* were realized, which were some filamentous fungi (*Rhizopus*, *Aspergillus*, etc.), yeasts (*Saccharomyces, Candida*, etc.), and bacteria (lactic acid bacteria, *Bacillus*. spp, etc.) (Zheng et al., 2012). Subsequently, the production technology of fortified *Daqu* based on functional isolates was also successfully developed (Li et al., 2019; Song et al., 2019). The quality and yield of fresh Baijiu were not only increased but also the abundance of some functional bacteria, such as *Caproiciproducens*, *Clostridium*, *Methanobacterium*, and *Methanosarcina* in PM, were enhanced when the fortified *Daqu* was once used (He et al., 2019c).

The activity of PM is closely related to the periods used unremittingly. Their contribution to the high quality of fresh Baijiu can be the overview as “the longer the pits unremittingly used, the higher quality baijiu yield” (Liu M. K. et al., 2017). It depended on the abundance of the unique functional microbiota in PM according to the results reported previously, and *Clostridia* and methanogenic archaea dominated on whether it was naturally domesticated or constructed with artificial pit mud (Chai et al., 2021). The mud pit was a unique micro-ecosystem inhabiting the functional microbiota, and the various metabolisms were performed synergistically by regulating based on the interaction of the interphase microbial community (Ding et al., 2015; Li et al., 2017). The evolution of the functional microbiota in the artificial PM was also significantly affected by the interaction of the interphase microbial community (Zhang et al., 2015). However, the contribution of fermented grains fermented with *Daqu* enriched with different microorganisms to the microbiota in the pit mud is not known.

The main purpose of the present study is to evaluate the effects of four kinds of *Daqu* on FG fermentation metabolism and PM domestication. Differences in community composition in different micro-ecosystems were investigated using high-throughput sequencing. Profiles of microbial metabolites and the accumulation of volatile compounds in FG fermentation were studied by HS-SPME-GC-MS. Moreover, evaluation of the relationship between the physicochemical properties of FG and PM, the dominant bacteria, and the accumulation of volatile compounds were also investigated to provide an insight into the mechanism of volatile compounds generated. A theoretical basis is laid for the regulation of FG functional microbial community and their metabolism and the acceleration of PM community evolution by applying *Daqu* enriched with different microbial communities.

## Materials and Methods

### Experimental Process and Sampling

Samples of sorghum, rice husk, fermented grains, *Daqu* [traditional *Daqu* (DZ), Qianghua *Daqu* (QH), Taikong *Daqu* (TK), and HH *Daqu* (QH:DZ = 1:1)] and 1-year artificial pit mud (PM0) were obtained from Lu Zhou Lao Jiao Co, Ltd. (Luzhou city, Sichuan Province, China), a famous Chinese liquor enterprise. QH *Daqu* was inoculated with *Bacillus velezensis* and *B. subtilis* and produced according to the manufacturing process described by He et al. (2019b). TK *Daqu* was manufactured *via* inoculating *muqu* at 1% ratio by weight of wheat. The *muqu* was expansion cultured by batch *via* inoculating traditional *Daqu* powder stayed in the capsule of Shenzhou 11 spacecraft for a month in space (Chen and Zhou, 2021). All standard chemicals used for quantification were mass spectrometry levels (> 97% purity) purchased from Sigma-Aldrich Co., Ltd. (Shanghai, China). Other reagents were purchased from China National Pharmaceutical Group Corporation (Shanghai, China).

The 5-L plastic containers (28 cm × 19 cm × 14 cm) lined with PM0 were used as the simulated small pit, and the bottom and surrounding thicknesses of the pit mud were 4 and 3 cm, respectively. The fermentation process was regulated according to the process operation specification ordained by the enterprise standard DB510500/T36-2016. Sorghum, a previous round of FG, and pre-cooked rice husks with a proportion of 1:4:0.2 were steamed for 1 h. When it was cooled below 40°C, the *Daqu* was added at the ratio of 15% (by dry weight of sorghum, called the initial FG). Subsequently, the initial FG was loaded into the simulated pit when cooled below 24°C, sealed by 4 cm thickness PM, and fermented for 60 days at 28 ± 2°C. Each group was composed of three biological parallel samples. At the end of fermentation, FG (DZ-FG, QH-FG, TK-FG, HH-FG) and PM (DZ-PM, QH-PM, TK-PM, HH-PM) samples were taken from the surrounding and central five points of the mud pit. These samples were mixed evenly and transferred to sterile polyethylene bags, and then stored at -80°C for analysis of physicochemical properties, metabolic components, and microbial community diversity.

### Detection of Physicochemical Properties

Moisture was determined by the gravimetric method by drying FG and PM samples at 105°C for 4 h. The pH of the samples was measured with a pH meter (PHS3C, Dapu Instruments Co., LTD., Shanghai, China) after mixing with carbon dioxide-free water at a ratio of 1:9 (Tan et al., 2019). Titratable acidity and total acidity were determined by titration referred to the approaches described by He et al. (2019a), and titrated with 0.1 mol/L NaOH solution to the endpoint of pH 8.2. Starch and reduction sugar content was measured by the method described by Wu (Wu et al., 2013). The ethanol content of the fermented grains was determined by an alcoholmeter after distillation and the content of total ester was measured *via* the titrimetric method after saponification (Mu et al., 2022). The 5-g samples were diluted with 20 mL, 9.00 mM of H_2_SO_4_, ultrasonically treated at indoor temperature for 60 min, and then centrifuged at 4°C and 12000 × *g* for 15 min. The obtained supernatant was filtered through a 0.22-μm syringe filter (Nylon Acrodisc, Waters Co., Milford, MA, United States) before determining the organic acid concentration using high-performance liquid chromatography (HPLC) (Wang X. et al., 2018).

Volatile flavor metabolites in FG were determined by headspace solid-phase microextraction coupled with gas-phase mass spectrometry (HS-SPME-GC-MS). Volatile flavor metabolites were adsorbed on fiber coated with 50/30 μm DVB/CAR/PDMS (Supelco, Bellefonte, PA, United States), inserted into the injection port to desorb for 5 min, and soon analyzed by GC-MS (Thermo trace 1300 gas chromatography coupled with a TSQ 9000 mass spectrometer, Thermo Electron Corporation, Waltham, MA, United States). GC-MS conditional parameter setting is based on the method described by Zhang et al. (2016). HS-SPME extracts were injected in a 20:1 split mode and analyzed on the VF-WAX-MS capillary column (30 m × 0.25 mm i.d., 0.25 μm film thickness, Agilent, Santa Clara, CA, United States). The carrier gas was helium with a purity of 99.99%. GC-MS conditions were as follows: the starting temperature was 40°C (held for 5 min), and it increased to 100°C at a rate of 4°C/min and then heated to 230°C at a rate of 6°C/min for 10 min. The injection temperature and ion source temperature were 270 and 300°C, respectively, and the MS scan range was m/z 35–400. Peak identification with a matching degree greater than 800 from the NIST2017 mass spectrometry library was analyzed. Based on the peak area of the internal standard methyl octanoate, the content of the identified volatile compounds was calculated. The odor activity values were calculated with reference to the odor thresholds of volatile flavor substances described by Liu and Sun (2018) and heat map cluster analysis was performed for substances with OAV > 0.1.

### Determining the Difference in the Microbial Community Diversity Among Samples

Quantitative analysis of microorganisms in PM was performed utilizing fluorescence *in situ* hybridization (FISH), a method described by Ding et al. (2014). All oligonucleotide probes we used have been listed in Supplementary Table 3 and were synthesized with the dye Cy3 at the 5’ end by Sangon (Shanghai, China).

Samples of total genomic DNA were extracted with the Omega Mag-bind soil DNA kit (Omega Bio-Tek, Doraville, GA, United States). The concentration, purity, and integrity of the extracted DNA were determined using a NanoDrop ND-1000 spectrophotometer (Thermo Fisher Scientific, Waltham, MA, United States) and 1% agarose gel electrophoresis, respectively. Bacteria and fungi were amplified by PCR based on the V3–V4 hypervariable regions of the 16S rRNA gene and ITS1 region, respectively. PCR amplification products were purified by agarose gel electrophoresis and the target fragment was recovered. The DNA concentration of samples was detected by a fluorescence quantitative spectrophotometer (BioTek, FLX800T, United States). Isoconcentration mixed samples were sequenced using paired-end 2 × 300 bp sequencing Illumina MiSeq platform by Shanghai Paesano Biotechnology Co., Ltd. (He et al., 2019a).

The original sequence was processed by qiime dada2 denoise-paired software.^1^ The high-quality sequences with 97% sequence identity were clustered into operational taxonomic units (OTUs) by UCLUST (Edgar, 2010^2^). Chimeric sequences and low-quality sequences including reading length less than 150 bp, mean base quality score less than 20, and mononucleotide repeats over 8 bp were filtered out. Finally, Silva (Release 13.8^3^) and UNITE (Release 8.0^4^) databases were applied to classify each sequence of 16S and ITS genes, respectively (Mu et al., 2022).

### Statistical Analysis

Heatmap analysis was performed by the R software (package v3.2.0). The correlation coefficient between microbes and volatile flavor metabolites was visualized by Cytoscape (v3.9.0). Principal coordinate analysis (PCoA) was used to evaluate the similarity of different communities. Mantel test analyzes the relationship between physicochemical and FG and PM microbial communities. To predict the sources of microbial communities in PM samples, Source Tracker (v0.9.8) was used with the default parameters. The differences between the statistical significance (*p* < 0.05) and the means of samples were tested by one-way analysis of variance (ANOVA) using SPSS 19.0 software (SPSS Inc., Chicago, IL, United States). Excel 2020 and Origin 2021 software were used to plot and statistical analysis and the results were expressed as mean ± standard deviation.

### Data Availability

The raw sequencing data sets have been deposited in the NCBI Sequence Read Archive (SRA^5^) and are available under accession number: PRJNA805079.

## Results and Discussion

### Influence of Different *Daqu* on the Diversity of Microbial Communities

After removing the low-quality sequences, chimeric sequences, and PCR primers, effective sequences with 3599 bacterial OTUs and 1126 fungal OTUs were obtained by 97% sequence identity cutoff. The rarefaction curve of each sample demonstrated that the sequencing data were enough for subsequent analysis (Supplementary Figure 1), which indicated that the sequencing could represent the actual situation of the microbial community, and the sequencing quality was high. There were significant differences in the alpha diversity of the community among samples, both before and after fermentation. In the initial phase of fermentation, α-diversity index of the bacteria in DZ0-FG was the highest, followed by TK0-FG, but decreased in the ended phase. However, the fungi were the highest in TK0-FG, and there was no significant change between samples at the end of fermentation (Supplementary Table 1), which was consistent with previous reports (Hu et al., 2016). The variation trend of bacterial and fungal communities in PM showed a similar pattern to those of FG, but bacterial abundance increased in QH-PM and TK-PM, while the fungal abundance and diversity decreased slightly (Supplementary Table 2). Fungi are more resistant to environmental stresses since their cellular structures, as well as DNA structures, were more complex than bacteria, so they evolve slowly in a directed manner (Liu M. et al., 2017). Although the process of domestication is slow, QH and TK *Daqu* are more favorable for PM domestication than DZ and HH *Daqu*.

**FIGURE 1:**
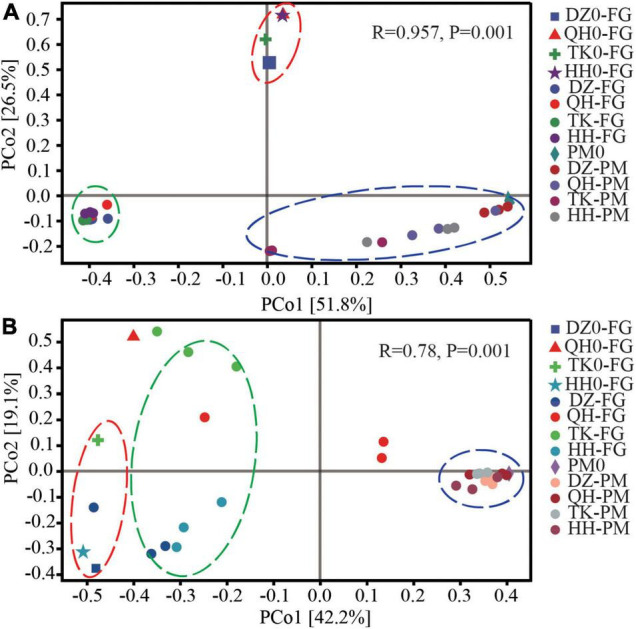
β-diversity of the microbial communities in FG and PM. PCoA analysis of FG and PM for bacterial **(A)** and fungal **(B)** communities. Principal compounds analysis (PCoA) based on the Bray–Curtis dissimilarity matrix of microbial communities. DZ0-FG, QH0-FG, TK0-FG, and HH0-FG are the FG at the beginning of fermentation. DZ-FG, QH-FG, TK-FG, and HH-FG are fermented grains after fermentation. PM0 is a new pit mud that has been artificially cultivated for 1 year. DZ-PM, QH-PM, TK-PM, and HH-PM are after fermentation.

Principal coordinate analysis analyses were performed using the Bray–Curtis algorithm to evaluate β-diversity in the microbiota in both FG and PM samples. These results demonstrated that the bacterial and fungal community profiles all could be divided into three clusters, including initial and ended FG, as well as another cluster for PM (Figure 1). In addition, cluster analysis showed that except for DZ *Daqu*, the remaining three *Daqu* altered the β-diversity of the community in both FG and PM. The changes in the distance between the initial and ended phases were also different in both PM and FG. For bacterial communities, the distance between PM and FG decreased to different degrees at the end of fermentation, and the change distance of PM was the most significant compared with that of FG for TK, followed by QH and HH, and almost no change in DZ. Principal coordinate axis one (PCo1) explained 51.8% of the variance in bacteria across all samples, and a significant difference (*P* < 0.01) was detected between the PCo1 scores for the FG and PM groups, which was also supported by ANOSIM (*R* = 0.957, *P* = 0.001) (Figure 1A). *Daqu* had a more significant effect on the fungal community in FG than bacterial, while PM clustered in the same cluster, and this was also confirmed by ANOSIM (*R* = 0.78, *P* = 0.001) (Figure 1B). The fungal community of *Daqu* had a strong disturbance effect on FG, while bacterial community succession was more conducive to the domestication of PM.

### Microbial Community Structure of Fermented Grain and Pit Mud

**FIGURE 2:**
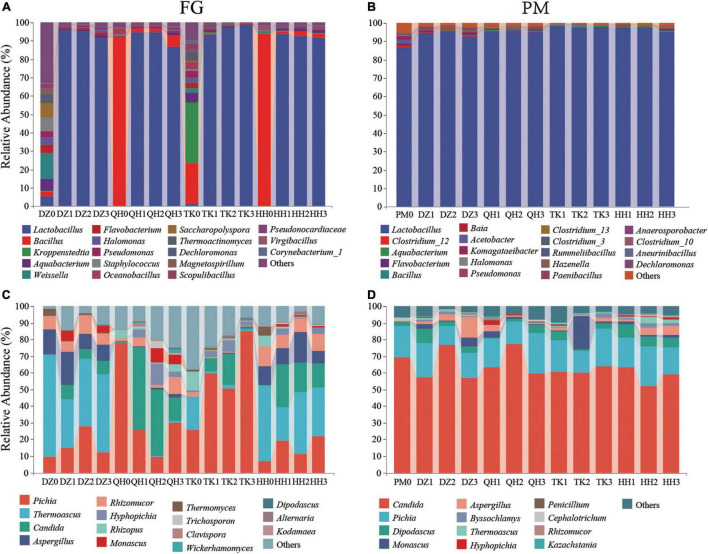
Distribution of microbial community structure during simulated fermentation. Different groups of the relative abundance of bacterial taxa were represented at the genus level (**A:** FG and **B:** PM). Different groups of the relative abundance of fungal taxa were represented at the genus level (**C:** FG and **D:** PM). Relative abundance of microorganisms > 1%. DZ0, QH0, TK0, and HH0 are the fermented grains at the beginning of fermentation. PM0 is a new pit mud that has been artificially cultivated for 1 year. The remaining samples correspond to the FG and PM of different *Daqu* fermentation systems.

The profile of the dominant community (relative abundance > 1%) at the genus level is shown in Figure 2. For bacterial communities, the abundance of *Weissella* was 13.73%, followed by *Saccharopolyspora* (7.51%) in the fermentation initial phase of DZ0-FG. After fermentation ended, the abundance of *Lactobacillus* was increased up to 94.14%, followed by *Aquabacterium*, *Halomonas*, and *Pseudomonas*, and the abundances were 1.47, 0.92, and 0.82%, respectively. Besides, the abundance of *Kroppenstedtia* and *Bacillus* predominated 32.95 and 21.94% in the TK0-FG, respectively. And then, the abundances of *Flavobacterium*, *Halomonas*, *Pseudomonas*, *Saccharopolyspora*, *Thermoactinomyces*, *Dechloromonas, and Corynebacterium_1* were also higher in the initial phase in both DZ0-FG and TK0-FG, although not dominated. After fermentation ended, *Kroppenstedtia*, *Flavobacterium*, and *Halomonas* dominated in TK-FG, in addition to *Lactobacillus*, while *Flavobacterium* and *Halomonas* co-dominated in both TK-FG and DZ-FG. The abundance of *Bacillus* was 91.82 and 93.55% in QH0-FG and HH0-FG, respectively. In the end, *Bacillus* also had a definite advantage, especially in the QH-FG group (Figure 2A). It is perhaps related to the uniqueness of the community composition of both QH *Daqu* and HH *Daqu*, as the former is fortified by *Bacillus* (He et al., 2019a). As reported by Wang et al. (2017), the community constituents in the initial fermentation grains were mainly closely related to that of *Daqu* due to *Huizao*, the last batch of FG had been cooked for more than 1 h. The abundances of *Weissella*, *Staphylococcus*, *Saccharopolyspora*, *Pseudonocardiaceae*, and *Corynebacterium_1* significantly reduced, while *Lactobacillus* dominated in all FG when fermented. L*actobacillus* propagated rapidly in the initial phase, and some metabolites, such as organic acids (especially acetic acid and lactic acid), inhibited the reproduction of some other bacteria, so that dominated the fermentation when the traditional *Daqu* was used (Hu et al., 2016). Besides, lactic acid favored the enrichment of the caproate-producing bacteria (Konopka et al., 2015). *Lactobacillus* dominated was 86.65%, followed by *Clostridium_12* in the initial phase of PM0. Lactic acid secreted by lactic acid bacteria inhibited certain microorganisms (Li et al., 2016). And that, the abundance of *lactobacillus*, an acid-tolerant facultative anaerobic microorganism, is closely related to the acidity or pH of PM and has an important stress effect on PM ecology. When fermentation finished, the abundance of *Lactobacillus* in TK-PM increased slightly and the abundance of *Bacillus* in QH-PM increased significantly in line with the results of FG, which is tentatively inferred to be the result of the effect of FG. However, there was almost no change in DZ-PM and QH-PM, and only *Aquabacterium* and *Halomonas* increased (Figure 2B).

As shown in Figure 2C, *Pichia* in both QH0-FG and TK0-FG dominated, their abundance was 78.11 and 25.52%, individually, and were consistent with our previous results reported (He et al., 2019a,Chen and Zhou, 2021). However, *Thermoascus* dominates in DZ0-FG and HH0-FG, followed by *Aspergillus* and *Rhizomucor*, but *Thermoascus* decreased and remained dominant in both DZ-FG and HH-FG, while the abundance of *Pichia* increased in TK-FG. In contrast, the dominant fungus shifted from *Pichia* to *Candida* in QH-FG. A similar result was also reported by Xiao et al. (2021). As shown in Figure 2D, there were no significant differences in the fungal community diversity among the PM samples despite the various type of *Daqu* used. The analysis results were consistent with the PCOA analysis described earlier. For the fungal genus, the dominant fungus *Candida* in PM was a strong acetic acid producer and *Pichia* was a contributor of several esters and aromatic components (Li et al., 2011). The result suggested that the perturbation effect on PM fungi was significantly lower than that of bacteria.

### Domestication of Pit Mud by Fermented Grain Microbial Succession

The profile of PM community composition was quantified based on FISH technology to further illustrate the effect of FG on PM community succession (Table 1). The results of the DAPI probe showed an increase in the total number of PM bacteria at the end of fermentation using TK *Daqu* and QH *Daqu*, with an increase of about 30%. Among them, the number of eubacteria and archaea all increased, and the increase of QH-PM was the largest, which was 1.42 times and 1.27 times that of DZ-PM, respectively. However, QH *Daqu* contributed to enhancing the abundance of *Methanosarcinales* but reduced that of *Methanobacteriales* and *Methanomicrobiales*. TK *Daqu* contributed to increasing the abundance of *Methanobacteriales* and *Methanosarcinales*. The proportion of hydrogenotrophic methanogens was roughly above 50% (51.97 and 52.94%) in the corresponding PM, which was consistent with that of matured PM (Xiao et al., 2021). These results not only validated the results by amplicon sequencing but also confirmed the regulation of bacterial community structure by TK *Daqu* and QH *Daqu*, especially *Archaea*. The number of archaea was used as an important indicator of matured PM or high-quality PM, which have a high activity of functional microbiota (Wu et al., 2015). The co-occurrence network showed that *Methanosarcinales* were positively related to the occurrence of *Bacillus*, *Methanobacteriales* were positively related to *Weissella*, and *Staphylococcus, Kroppenstedtia*, and *Methanosarcinales* were positively related to *Lactobacillus* and *Corynebacterium_*1 (Supplementary Figure 2). The increase in archaea led to an enhanced ability to utilize H_2_, CO_2_, and acetic acid, which was beneficial to maintaining the stability of acidity in Baijiu fermentation niches.

**TABLE 1:** Concentration of different microorganisms detected by FISH in pit muds (*10^9^cells/g).

Probes	DZ-PM	QH-PM	TK-PM	HH-PM
DAPI	5.16 ± 0.11^c^	6.72 ± 0.19^a^	6.68 ± 0.09^a^	5.66 ± 0.16^b^
EUB338	1.72 ± 0.09^c^	2.44 ± 0.10^a^	2.11 ± 0.17^b^	2.20 ± 0.08^b^
ARCH915	3.16 ± 0.07^c^	4.00 ± 0.07^a^	3.56 ± 0.09^b^	3.46 ± 0.13^b^
MB311	0.07 ± 0.04^ab^	0.05 ± 0.02^ab^	0.11 ± 0.04^a^	0.04 ± 0.04^b^
MG1200b	2.47 ± 0.10^a^	1.93 ± 0.10^b^	1.42 ± 0.15^c^	2.65 ± 0.10^a^
MSMX860	0.50 ± 0.06^d^	1.83 ± 0.06^a^	1.36 ± 0.14^b^	0.69 ± 0.04^c^

*Values with different letters within a row are significantly different statistically (P<0.05).*

**FIGURE 3:**
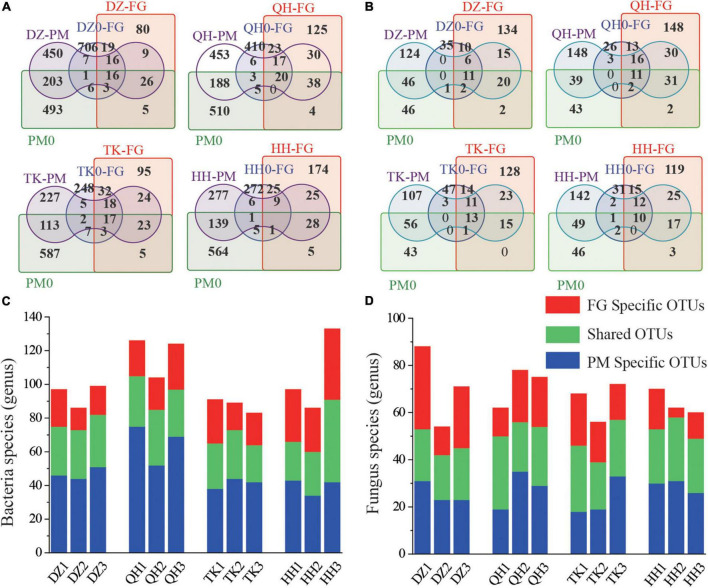
Bacterial and fungal migration between the different microbial habitats in the Baijiu fermentation pit. Venn diagram of OTUs distribution based on bacterial **(A)** and fungal **(B)** in FG and PM. The OTUs values of FG (DZ-FG, QH-FG, TK-FG, HH-FG) and PM (DZ-PM, QH-PM, TK-PM, HH-PM) after fermentation were the averages of three parallel groups. Histogram showing the number of shared OTUs for FG and PM samples of fermentation finished. **(C)** Bacterial and **(D)** Fungal.

Tracking individual OTUs in different samples revealed widespread species migration and colonization between the FG and PM communities (Figure 3). At the initial phases of fermentation, DZ0-FG, QH0-FG, TK0-FG, HH0-FG, and PM0 shared OTUs of 6, 5, 7, and 5, respectively, and when the fermentation finished, they increased to 9, 30, 24, and 25, respectively (Figure 3A). In the fungal community, there were few contributing genera between FG and PM in the initial phase, while the number of contributing genera increased significantly when ended (Figure 3B). The evolution of community structure and intermigration has led to a significant increase in the number of shared genera. Microbial interactions have been frequently proposed as potential drivers of microbiome diversity and thereby determining the performance of their communities. The microbial interaction in a mixed fermentation system is an important aspect of investigating the fermentation process since it is related to the fluctuation of microorganisms and directly affects the microbial metabolism over the process (Liu M. et al., 2017). Specific bacterial OTUs were lower for TK-PM and HH-PM than for DZ-PM and QH-PM, while there were no significant differences for fungi (Figures 3C,D). Meanwhile, among the facultative anaerobes detected in FG, some genera (e.g., *Lactobacillus, Bacillus, Halomonas, Flavobacterium, Pseudomonas*, and *Dechloromonas*) could also be discovered in PM (Figure 2). These prokaryotes were also detected in PM in previous research, which should be the result of FG migration and colonization (Ding et al., 2015). PM communities are more stable than FG, indicating that the evolution of the community in PM is a slow process. We used the Bayesian probability Source Tracker tool to further predict the sources of microbes found in PM. FG contributed 67.84, 74.75, 85.01, and 79.51% to the bacterial communities of DZ-PM, QH-PM, TK-PM, and HH-PM, respectively, but only 31.12, 34.05, 14.50, and 20.00% in the initial stages of the corresponding samples (Supplementary Figure 3A). For fungal communities, FG contributed 5.07, 16.44, 10.42, and 9.84% to the DZ-PM, QH-PM, TK-PM, and HH-PM, respectively (Supplementary Figure 3B). It can be seen that exogenous microorganisms have a significant effect on the bacterial community of PM, especially by TK *Daqu*, and have been validated by the results of the β-diversity analysis.

### Correlation Between Microbial Communities and Physicochemical Parameters

**FIGURE 4:**
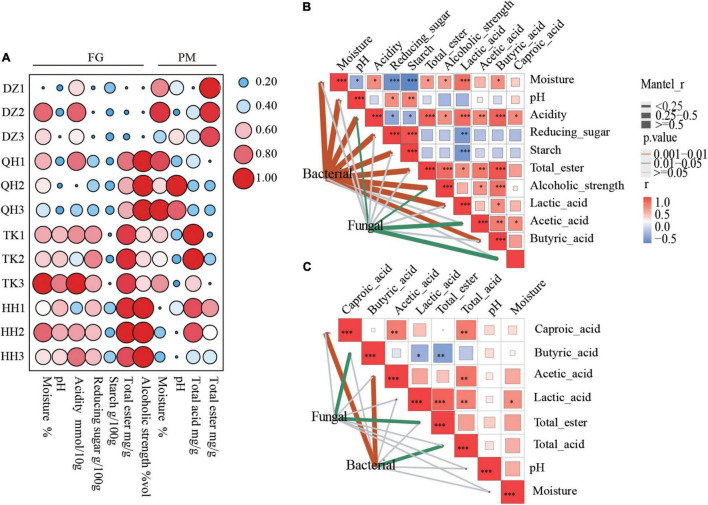
Changes in the physical and chemical properties of FG and PM during fermentation **(A)**. Environmental drivers of FG **(B)** and PM **(C)** microbial community composition. Pairwise comparisons of environmental factors are shown, with a color gradient representing Spearman’s correlation coefficients. Bacterial and fungal community composition was related to each environmental factor by partial (geographic distance-corrected) Mantel tests. Edge width corresponds to the Mantel’s r statistic for the corresponding distance correlations, and edge color denotes the statistical significance based on 9,999 permutations.

The quality and yield of NXBJ are closely related to the bioactive biological activity and pit age of PM, and changes in FG physicochemical parameters are also important factors (Xiao et al., 2019; Zhang et al., 2020). The changes in trends of physicochemical properties that resulted from the different types of *Daqu* can be observed in FGs and PMs (Figure 4A). The contents of starch and reducing sugar in FGs decrease significantly at the end of fermentation, while alcoholic strength shows an opposite trend. It is noteworthy that QH-FG has the lowest starch utilization ratio but the alcoholic strength was the highest. The alcoholic strength in TK-FG was significantly higher than that in DZ-FG but was lower than QH-FG and HH-FG. It was perhaps related to the abundance of *Bacillus* in the fermented grain fermentation initial phase of TK0-FG, which was significantly higher than that in that of DZ0-FG, but lower than in that of QH0-FG and HH0-FG (Figure 2A). A higher abundance of *Bacillus* was observed in QH *Daqu*, which resulted in improving the saccharification and ethanol fermentation (He et al., 2019a). The change of the physiochemistry parameters in PM was weaker than those in FG, which were consistent with the pattern of microbial changes and reported by Song et al. (2017). There were significant differences among these samples, whether FG or PM as well as between the two, for the main organic acid (lactic acid). For example, the increment of DZ-FG was significantly higher than the remaining samples, and similar results were observed as well as for PM as shown in Supplementary Figures 4A,B. Organic acids, especially lactic acid and acetic acid, were deposited with water into the substratum, and the pH decreased, which was reported by Zheng et al. (2014).

The remarkable divergence in the structural succession of microbial communities was mainly responsible for the differences in chemical properties. Bacterial and fungal community composition was related to each environmental factor by Mantel tests. FG bacteria were strongly correlated with most environmental factors (Figure 4B) and PM bacteria were significantly correlated with total acid, especially acetic acid and hexanoic acid (Figure 4C). Related research results also indicated that bacterial community succession was significantly correlated with the dynamics of process parameters, such as temperature, moisture, and ethanol content in FG over the process (Zhang et al., 2021b). Environmental parameters had less effect on fungi than bacteria, and PM fungi were significantly correlated with total esters and butyric acid. There was a weak correlation between fungi and acidity, acetic acid, capric acid, and alcoholic strength in FG. These results demonstrated that the flavor precursors depended on the community structure and its metabolic processes. Related studies have confirmed that butyric and capric acid producers were mainly derived from microbes of PM (Chai et al., 2019) and that the latter abundance was closely related to pit age (Liu M. et al., 2017).

### Correlation of Dominant Microbes With Flavor Compounds

**FIGURE 5:**
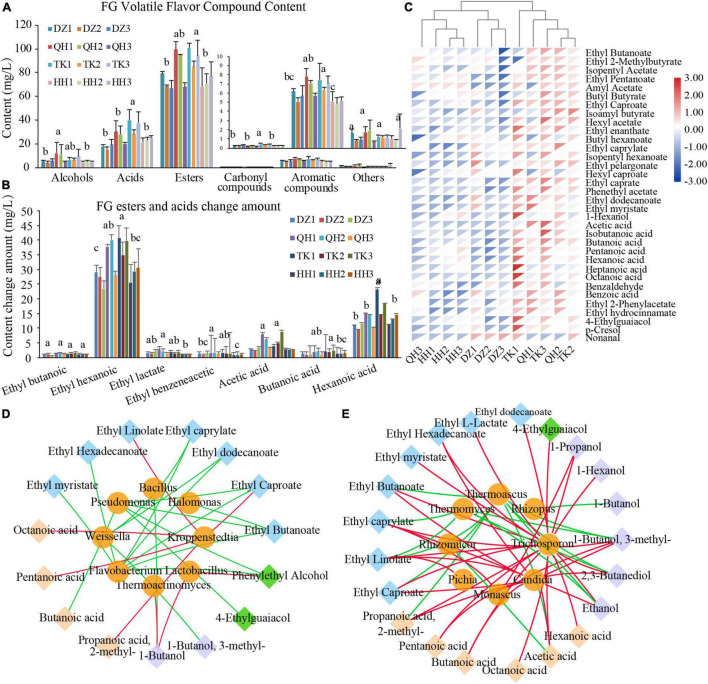
Analysis of the difference between volatile flavor metabolites and microbial correlation in fermented grains. **(A)** Content of different classes of volatile flavor metabolites. **(B)** Changes in the content of major volatile flavor metabolites acids and esters in fermented grains before and after fermentation. **(C)** Heatmap of volatile flavor metabolites in fermented grains. The OAV values in different samples were calculated with reference to the odor thresholds described by Liu et al. The volatile flavor metabolites with OAV > 0.1 were then selected for clustering analysis. DZ-FG (DZ1, DZ2, DZ3), QH-FG (QH1, QH2, QH3), TK-FG (TK1, TK2, TK3), and HH-TK (TK1, TK2, TK3). The correlation of bacterial **(D)** and fungi **(E)** communities with flavor compounds by network correlation analysis. The color of the lines corresponds to positive (red) or negative (green) correlations. The thickness of lines is proportional to the value of Spearman’s correlation coefficient.

A total of 100 volatile metabolites were identified by HS-SPME-GC-MS in FG samples, and these metabolites were distributed across six chemical classes: 62 esters, 12 alcohols, 9 acids, 9 aromatics, 4 carbonyl compounds, and 4 others. Esters were the most abundant in the identified volatiles of sample FG, accounting for 59.95 to 67.72%, followed by alcohols and acids (Figure 5A). The most important odor characteristics in NXBJ are fruity, sweet, and floral, and these aromas are mainly contributed by esters, accounting for 75∼95% of volatile metabolites. Therefore, their contents and proportion are important indexes to evaluate Chinese liquor quality (Fan et al., 2018a). Among them, some volatiles has been identified as important aroma substances of NXBJ, such as ethyl butyrate, ethyl caproate, ethyl lactate, ethyl phenylacetate, acetic acid, butyric acid, caproic acid, and especially ethyl hexanoate (Zhao et al., 2018). It was also recognized that hexanoic acid and ethyl caproate are skeleton components distinguishing other types of Baijiu (Zhao et al., 2018). The dominant volatiles in TK-FG were significantly higher than the remaining samples, especially ethyl caproate, acetic acid, and caproic acid have significant content advantages and greatly affect the flavor profiles of FG (Figure 5B). Heat map analysis of volatile components with OAV > 0.1 further showed that TK-FG had a high content of active flavor components, while TK-FG and QH-FG clustered closely due to similar composition and HH-FG and DZ-FG clustered together with low flavor content and similar structure (Figure 5C).

Changes in volatile flavor-related metabolites may be mediated by the community diversity and their metabolism. Therefore, revealing the potential correlations between microbes and metabolites is crucial to optimize the process, as well as promote the improvement of the new artificial PM activity. Network correlation analysis was applied to investigate the correlation between microbes and flavor compounds of FG. Spearman’s correlation coefficients (| ρ| > 0.7, *P* < 0.05) of dominant genera (relative abundance > 1%) and volatile flavor metabolites constructed correlation network. As shown in Figure 5D, *Kroppenstedtia* was positively correlated with most volatile acids, ethyl caproate, and ethyl linolenic acid, while *Lactobacillus* was positively correlated with benzyl alcohol and butanol. As a dominant microbe in TK *Daqu*, *Kroppenstedtia* can significantly increase the content of main volatile components such as esters, alcohols, acids, pyrazines, and so on, and its esterification capacity was 34.41% higher than that of DZ *Daqu*, which was beneficial to promote the formation of esters (Chen and Zhou, 2021). *Lactobacillus* plays a key role in the metabolism of volatiles and their precursors (Du et al., 2020) and can promote the accumulation of several esters (Li et al., 2020). *Trichosporon* and *Candida* were positively correlated with ester components, *Monascus* was positively correlated with ethyl caprylate, propanol, and 3-methyl-butanol, and *Thermoascus* and *Thermomyces* were negatively correlated with esters and alcohols (Figure 5E). However, the abundance of *Thermoascus* and *Thermomyces* decreased at the end of fermentation. Solid-state fermentation is a spontaneous fermentation process with a succession of microbial communities, and the accumulation of metabolites can affect the diversity and structure of the microbial community (Li et al., 2011). Likewise, the formation of volatile compounds lies in the synergy metabolism by the functional microbiota involved (Fan et al., 2018a). The volatile compounds were key drivers of community aggregation in the food industry (Zhang et al., 2021a). During the process, the difference in various volatile compounds produced by the unique microbiota endowed the distinct style (Qun et al., 2014).

## Conclusion

In conclusion, the difference in microbiota among *Daqu*’s not only affected the FG community diversity and differences in metabolites but also the biological activity of PM, especially bacteria microbiota. TK *Daqu* contributed to the content enhancement of ethyl hexanoate, one of the key flavor compounds, while the yield was enhanced when QH *Daqu* was used. HH *Daqu* contributes to a higher yield but the contribution of volatile flavor metabolites was much lower than the two formers. FG used TK *Daqu* which was more likely to disturb the bacterial community structure of PM. These results laid an important foundation for optimizing the strategy improving the yield and quality of Baijiu and accelerating the evolution of functional microbiota in new PM based on the distinctive microbiota in *Daqu*.

## Data Availability Statement

The datasets presented in this study can be found in online repositories. The names of the repository/repositories and accession number(s) can be found in the article/Supplementary Material.

## Author Contributions

RZ and JH conceived and designed the study. FM performed the experiments, analyzed the data, and wrote the manuscript. HQ, SZ, XC, and CQ provided financial support and sample collection. All authors read and approved the manuscript.

## Conflict of Interest

HQ, SZ, XC, and CQ was employed by Lu Zhou Lao Jiao Co., Ltd. The remaining authors declare that the research was conducted in the absence of any commercial or financial relationships that could be construed as a potential conflict of interest.

## Publisher’s Note

All claims expressed in this article are solely those of the authors and do not necessarily represent those of their affiliated organizations, or those of the publisher, the editors and the reviewers. Any product that may be evaluated in this article, or claim that may be made by its manufacturer, is not guaranteed or endorsed by the publisher.

## Footnotes

1
https://github.com/QIIME2/q2-feature-classifier

2
https://github.com/gcybis/Uclust

3
http://greengenes.secondgenome.com/

4
https://unite.ut.ee/

5
http://www.ncbi.nlm.nih.gov/bioproject/805079
