# Supplementary material for: Effects of Different Daqu on Microbial Community Domestication and Metabolites in Nongxiang Baijiu Brewing Microecosystem
Source: Front Microbiol. 2022 Jun 30;13:939904. doi: 10.3389/fmicb.2022.939904 (PMC9279870; doi:10.3389/fmicb.2022.939904)
Supplement: Supplementary file 1 [file Data_Sheet_1.docx]

Supplementary Material

# Supplementary Tables

**Supplementary Table S1.** Richness and diversity indexes of the microbial community in fermented grains.

| Sample (FG) | Diversity index | | | | Abundance index | | | | | |
| --- | --- | --- | --- | --- | --- | --- | --- | --- | --- | --- |
|  | Shannon | | Simpson | | Chao1 | | Observed species | | |  |
|  | Bacteria | Fungi | Bacteria | Fungi | Bacteria | Fungi | | Bacteria | Fungi | |
| **DZ0** | **6.43** | **2.15** | **0.97** | **0.60** | **781.51** | **66.16** | | **777.20** | **65.80** | |
| DZ1 | 1.48 | 3.54 | 0.52 | 0.85 | 102.12 | 110.05 | | 97.80 | 109.90 | |
| DZ2 | 1.56 | 2.82 | 0.53 | 0.77 | 82.14 | 58.93 | | 73.10 | 58.70 | |
| DZ3 | 2.00 | 3.04 | 0.57 | 0.75 | 127.27 | 97.05 | | 117.30 | 97.00 | |
| **QH0** | **4.35** | **2.04** | **0.84** | **0.45** | **483.44** | **71.05** | | **474.10** | **70.50** | |
| QH1 | 1.69 | 3.32 | 0.53 | 0.77 | 131.11 | 119.84 | | 118.40 | 116.40 | |
| QH2 | 1.80 | 3.71 | 0.55 | 0.82 | 151.60 | 127.73 | | 128.70 | 127.70 | |
| QH3 | 2.35 | 4.87 | 0.62 | 0.91 | 160.43 | 123.00 | | 149.50 | 122.90 | |
| **TK0** | **5.39** | **3.90** | **0.95** | **0.88** | **336.88** | **89.05** | | **331.90** | **89.00** | |
| TK1 | 1.69 | 2.84 | 0.53 | 0.66 | 186.08 | 112.87 | | 165.70 | 112.30 | |
| TK2 | 1.33 | 3.24 | 0.49 | 0.74 | 92.49 | 103.76 | | 85.90 | 102.70 | |
| TK3 | 1.18 | 1.73 | 0.48 | 0.36 | 76.33 | 79.03 | | 70.90 | 78.60 | |
| **HH0** | **3.07** | **3.04** | **0.71** | **0.76** | **346.97** | **73.80** | | **329.00** | **72.70** | |
| HH1 | 1.44 | 3.74 | 0.47 | 0.87 | 111.60 | 108.43 | | 101.40 | 107.40 | |
| HH2 | 1.69 | 3.01 | 0.52 | 0.79 | 150.00 | 87.66 | | 128.10 | 86.00 | |
| HH3 | 1.80 | 3.76 | 0.53 | 0.87 | 215.06 | 101.36 | | 192.80 | 100.50 | |

**Supplementary Table S2.** Richness and diversity indexes of the microbial community in pit mud.

| Sample (PM) | Diversity index | | | | Abundance index | | | | | |
| --- | --- | --- | --- | --- | --- | --- | --- | --- | --- | --- |
|  | Shannon | | Simpson | | Chao1 | | | Observed species | | |
|  | Bacteria | Fungi | Bacteria | Fungi | Bacteria | Fungi | Bacteria | | Fungi |  |
| **PM0** | **3.07** | **2.61** | **0.71** | **0.60** | **346.97** | **131.84** | **329.00** | | **129.60** |  |
| DZ1 | 1.44 | 3.31 | 0.47 | 0.72 | 111.60 | 136.82 | 101.40 | | 136.40 |  |
| DZ2 | 1.69 | 2.10 | 0.52 | 0.48 | 150.00 | 97.20 | 128.10 | | 96.20 |  |
| DZ3 | 1.80 | 3.19 | 0.53 | 0.72 | 215.06 | 113.57 | 192.80 | | 112.30 |  |
| QH1 | 2.93 | 3.02 | 0.49 | 0.65 | 791.51 | 141.51 | 749.30 | | 138.00 |  |
| QH2 | 1.86 | 2.12 | 0.37 | 0.47 | 435.54 | 125.08 | 364.60 | | 122.80 |  |
| QH3 | 1.73 | 2.92 | 0.33 | 0.68 | 416.18 | 141.11 | 376.30 | | 139.90 |  |
| TK1 | 2.07 | 3.08 | 0.42 | 0.67 | 457.01 | 116.81 | 398.30 | | 116.20 |  |
| TK2 | 2.35 | 2.43 | 0.59 | 0.63 | 352.12 | 96.80 | 303.50 | | 96.20 |  |
| TK3 | 2.50 | 2.76 | 0.58 | 0.64 | 448.95 | 125.99 | 383.70 | | 124.70 |  |
| HH1 | 1.91 | 3.05 | 0.38 | 0.66 | 467.37 | 134.47 | 405.30 | | 133.90 |  |
| HH2 | 2.45 | 3.44 | 0.67 | 0.74 | 267.60 | 142.19 | 238.10 | | 140.20 |  |
| HH3 | 2.48 | 3.15 | 0.71 | 0.68 | 249.16 | 118.14 | 202.00 | | 117.80 |  |

**Supplementary Table S3.** Oligonucleotide probes were used in the present experiment.

| Probes | Target microbes | GenBank （5’-3’） |
| --- | --- | --- |
| EUB338 | Domain bacteria | GCTGCCTCCCGTAGGAGT |
| Arch915 | Archaea | GTGCTCCCCCGCCAATTCCT |
| MB311 | *Methanobacteriales* | ACCTTGTCTCAGGTTCCATCTCC |
| MG1200 | *Methanomicrobiales* | CRGATAATTCGGGGCATGCTG |
| MSMX860 | *Methanosarcinales* | GGCTCGCTTCACGGCTTCCCT |

##
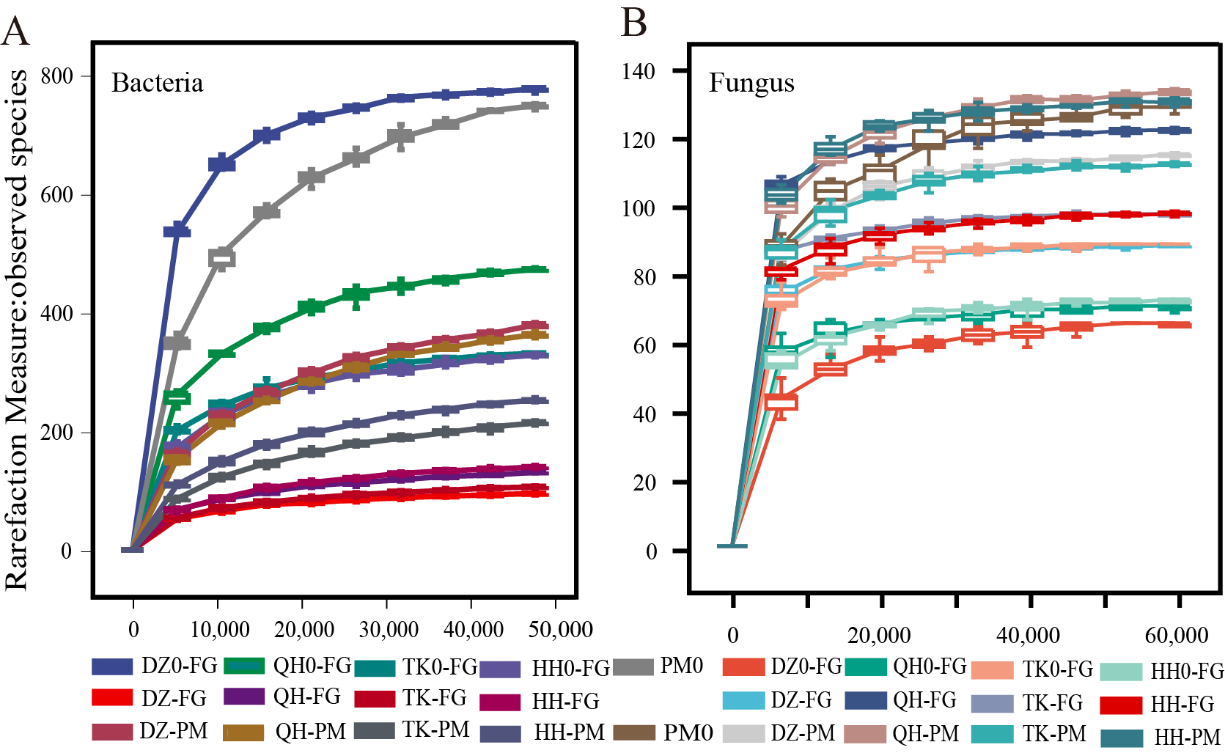
Supplementary Figures

##
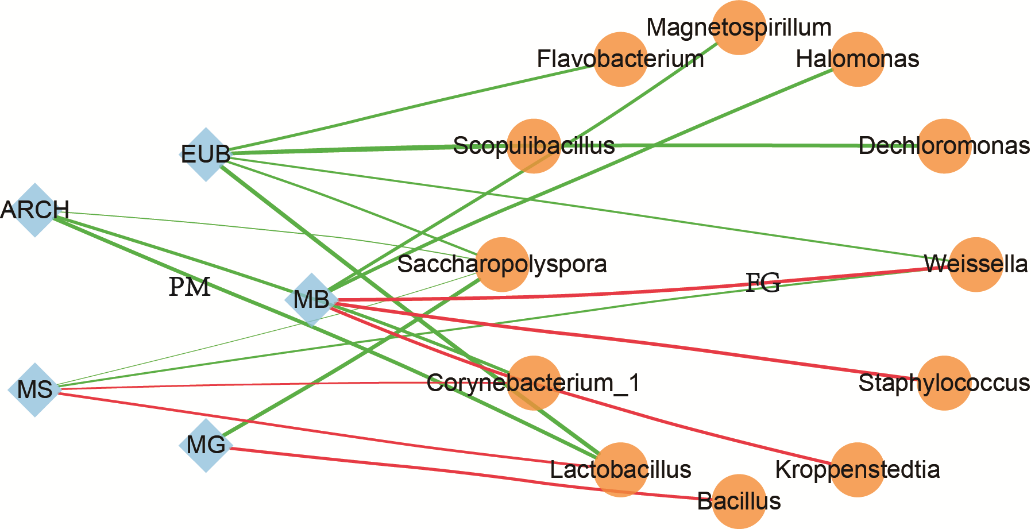
Supplementary Figure 1. Sparse curve of alpha diversity index of bacteria (A) and fungi (B) in fermented grains and pit mud samples.

**Supplementary Figure S2.** Correlation analysis between bacteria of fermentation grains and pit mud microorganisms. The color of the lines corresponds to positive (red) or negative (green) correlation. The thickness of lines is proportional to the value of Spearman’s correlation coefficient.


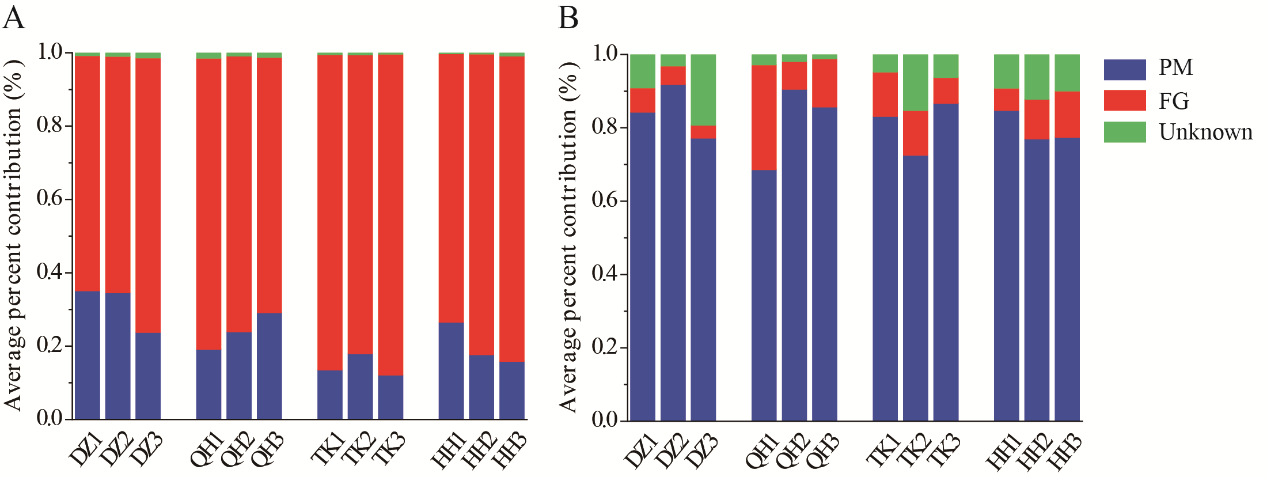
**Supplementary Figure S3.** Source Tracker results highlight the percentages of inferred sources of bacterial (A) and fungal (B) communities in pit mud.

**
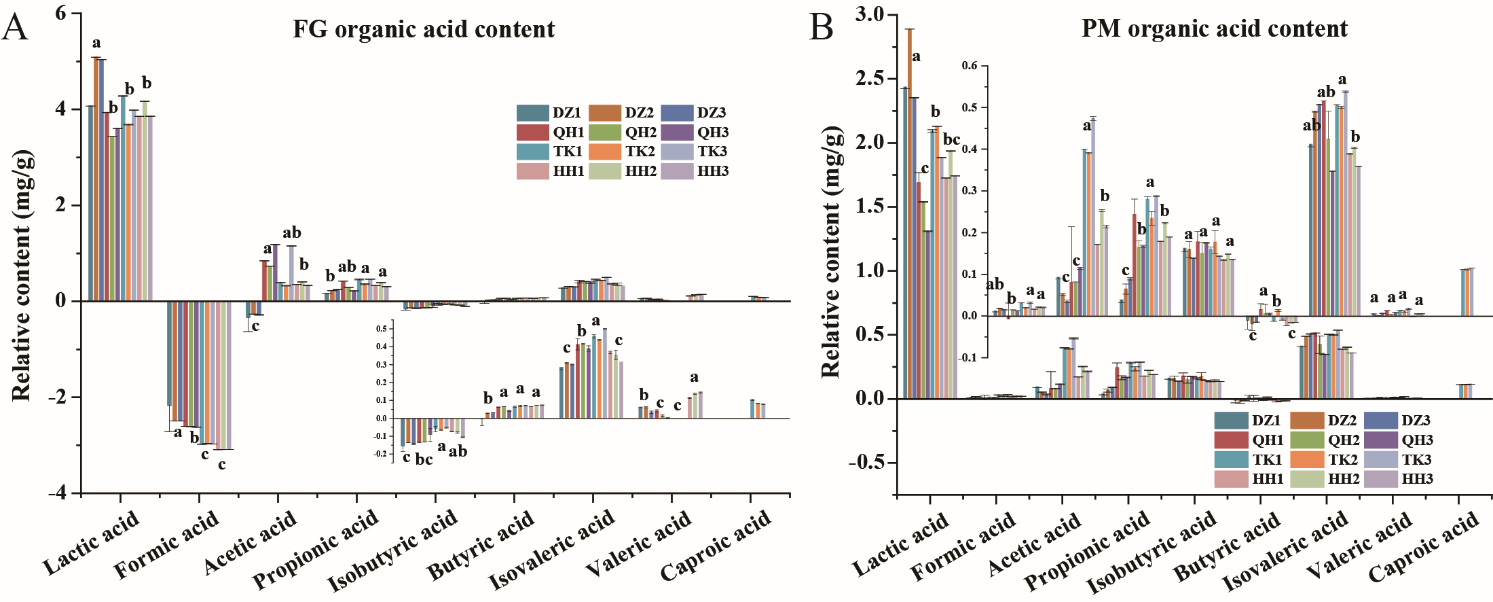
Supplementary Figure S4.** The change of organic acids of the fermented grains (A) and pit mud (B) in the simulating fermentation. Different letters represent significant differences (*P <* 0.05).
